# Mechanical considerations for deep bite correction with clear aligners-research article

**DOI:** 10.6026/973206300220806

**Published:** 2026-02-28

**Authors:** Kanchan Sharma, Shruti Gurjar, Pooja Pooja, Chitra Agarwal

**Affiliations:** 1 Department of Orthodontics and Dentofacial Orthopaedics, Awadh Dental College and Hospital Jamshedpur Jharkhand, India; 2 Consultant Orthodontist, Dentteeth Clinic, E-9 Ratlam Kothi, Geeta Bhawan, Indore, Madhya Pradesh, India; 3 Department of Orthodontics and Dentofacial Orthopaedics, Nims Dental College and Hospital, Jaipur, Rajasthan, India

**Keywords:** Deep bite, clear aligners, orthodontic biomechanics, incisor intrusion, clinical research

## Abstract

Deep bite malocclusion is a frequent orthodontic problem, and its correction with clear aligners remains biomechanically challenging due to limitations in vertical movement predictability. This study evaluated mechanical considerations and clinical outcomes of deep bite correction using clear aligner therapy in 30 patients. Pre- and post-treatment records were assessed for overbite reduction, incisor intrusion, molar extrusion, attachment protocols, staging design, and treatment duration. A significant overbite reduction was observed (5.5 ± 0.7 mm to 2.3 ± 0.6 mm), achieved mainly through maxillary (1.7 ± 0.4 mm) and mandibular incisor intrusion (1.4 ± 0.3 mm) with minimal molar extrusion (0.6 ± 0.2 mm). Clear aligner therapy was found to be effective for deep bite correction when proper biomechanical planning ensures controlled anterior intrusion and posterior vertical stability.

## Background:

Deep bite malocclusion is a common vertical discrepancy characterized by excessive vertical overlap of the maxillary and mandibular incisors. Clinically, it may be associated with functional disturbances, compromised esthetics, incise wear and periodontal problems. Correction of deep bite remains one of the more challenging aspects of orthodontic treatment because it requires precise control of vertical tooth movement while maintaining overall occlusal stability and facial harmony [1, 2]. Conventionally, deep bite correction has been achieved using fixed orthodontic appliances through various biomechanical approaches such as anterior bite planes, intrusion arches, reverse curve of Spee mechanics and segmented arch techniques [3, 4-5]. These approaches aim to correct excessive overbite by incisor intrusion, posterior tooth extrusion, or a combination of both, depending on the patient's skeletal pattern and facial proportions. While effective, fixed appliance therapy often involves complex wire mechanics, increased chairside time and greater dependence on operator skill [6]. In recent years, there has been a growing demand for esthetic orthodontic treatment, leading to increased use of clear aligner therapy in routine clinical practice. Advances in digital treatment planning, aligner material properties and attachment design have expanded the scope of clear aligners beyond mild malocclusions to include more complex cases, such as those involving vertical discrepancies [7, 8]. As a result, clear aligners are now frequently considered as an alternative to fixed appliances for managing deep bite malocclusion. However, the biomechanics of deep bite correction using clear aligners differ fundamentally from those of conventional fixed appliances. Clear aligners rely on programmed staging of tooth movement, attachment geometry, aligner thickness and intimate appliance fit to deliver orthodontic forces. Unlike fixed appliances, which provide continuous force delivery, aligners exert intermittent forces that are highly dependent on patient compliance and the accuracy of digital treatment planning [9]. This raises concerns regarding the predictability of vertical tooth movements, particularly true incisor intrusion, with aligner therapy [10]. Previous clinical studies evaluating the effectiveness of clear aligners have reported variable outcomes in deep bite correction. While some authors have demonstrated satisfactory overbite reduction, others have noted discrepancies between planned and achieved tooth movements, especially in the vertical dimension [11, 12-13]. Factors such as attachment design, staging protocols, control of posterior extrusion and refinement strategies have been identified as critical determinants of treatment success. Despite these observations, there is limited clinical literature focusing specifically on the mechanical considerations involved in deep bite correction with clear aligners in real-world practice. From a clinical standpoint, understanding how different biomechanical strategies influence treatment outcomes is essential for case selection, treatment planning and improving predictability. Retrospective evaluation of treated cases can provide valuable insight into the actual mechanisms by which deep bite correction is achieved with clear aligners and help identify factors associated with successful outcomes. Therefore, it is of interest to evaluate the mechanical considerations involved in deep bite correction using clear aligner therapy and to assess the clinical outcomes achieved in patients treated with this modality.

## Materials and Methods:

## Study design and setting:

This study was designed as a retrospective clinical research study conducted in an orthodontic clinical setting. Patient records were reviewed after obtaining approval from the institutional ethics committee. As the study involved retrospective evaluation of existing clinical data, informed consent was waived.

## Sample selection:

Clinical records of patients treated with clear aligner therapy were screened. A total of 30 patients diagnosed with deep bite malocclusion and treated exclusively with clear aligners were selected for inclusion in the study.

## Inclusion criteria:

[1] Patients with deep bite malocclusion, defined as an overbite of ≥4 mm

[2] Patients treated solely with clear aligner therapy

[3] Availability of complete pre-treatment and post-treatment records

[4] Records including lateral cephalograms, intraoral photographs and digital dental models

[5] Patients who completed the planned course of aligner treatment

## Exclusion criteria:

[1] Patients with craniofacial anomalies or syndromes

[2] Patients who underwent orthognathic surgery

[3] Patients requiring adjunctive fixed orthodontic appliances

[4] Incomplete or poor-quality clinical records

## Clinical records and data collection:

The following clinical records were obtained and analyzed for each patient:

[1] Pre- and post-treatment lateral cephalograms

[2] Pre- and post-treatment intraoral photographs

[3] Digital dental models generated from aligner treatment planning software

[4] All measurements were carried out by a single examiner to eliminate inter-examiner variability.

[5] To assess intra-examiner reliability, records of 10 randomly selected patients were re-evaluated after a two-week interval.

## Outcome measures:

The primary outcome measure was the amount of overbite correction achieved following treatment. Overbite was measured in millimeters on lateral cephalograms and digital models.

## Secondary outcome measures included:

[1] Amount of maxillary incisor intrusion

[2] Amount of mandibular incisor intrusion

[3] Degree of molar extrusion

[4] Treatment duration

[5] Requirement for refinement stages

## Assessment of mechanical considerations:

Mechanical strategies employed during treatment were evaluated based on treatment records and aligner planning data. These included:

[1] Type and location of attachments used for anterior intrusion

[2] Staging protocols for vertical tooth movement

[3] Measures employed to control posterior vertical dimension

## Cephalometric analysis:

Cephalometric analysis was performed using standardized landmarks and reference planes. Linear measurements were recorded to the nearest 0.1 mm. Pre- and post-treatment cephalograms were traced and analyzed using digital cephalometric software under standardized conditions.

## Statistical analysis:

All data were tabulated and analyzed using statistical software. Descriptive statistics, including mean and standard deviation, were calculated for all parameters. Comparison between pre-treatment and post-treatment measurements was performed using a paired t-test. The level of statistical significance was set at p < 0.05.

## Results:

A total of 30 patients with deep bite malocclusion treated using clear aligner therapy were included in the study. All patients completed the planned course of treatment and complete pre- and post-treatment clinical records were available for analysis. Clear aligner therapy resulted in a statistically significant reduction in overbite. The mean pre-treatment overbite was 5.5 ± 0.7 mm, which reduced to 2.3 ± 0.6 mm at the end of treatment. The mean overbite reduction achieved was 3.2 ± 0.6 mm and this difference was statistically significant (p < 0.001) (Table 1). Cephalometric evaluation showed that deep bite correction was primarily achieved through intrusion of the anterior teeth. The mean maxillary incisor intrusion was 1.7 ± 0.4 mm, while mandibular incisor intrusion averaged 1.4 ± 0.3 mm. Posterior teeth showed minimal extrusion, with a mean molar extrusion of 0.6 ± 0.2 mm, indicating effective posterior vertical control during aligner therapy (Table 2). Attachments designed for vertical control were used in all cases. Controlled staging of vertical movements was incorporated into the treatment plans to improve predictability. The mean treatment duration was 14.1 ± 2.0 months. Refinement aligners were required in 8 out of 30 patients (26.7%) to achieve the desired overbite correction (Table 3).

## Discussion:

In the current clinical trial, there was statistically significant change in the overbite after the use of clear aligners. The extent of overbite reduction is similar to those reported earlier which showed that clear aligners can actually achieve clinically significant vertical changes in properly chosen cases [7, 15]. The fact that the current findings coincide with previous studies contributes to the clinical efficacy of aligner therapy in case of sufficient attention to the key biomechanical principles. One of the most important results of the present research was that deep bite correction was attained mainly with the effect of controlled maxillary and mandibular incision instead of the posterior extrusion. Incisor intrusion is deemed to be superior in patients whose lower anterior facial height gains are not desired [4, 6]. The current results compare with the existing biomechanical ideas and indicate that clear aligners, in case they are designed correctly, can recreate the principles used with fixed appliances. The force of incisor intrusion that was reported in this research paper can be explained by the progress of attachment design and computerized staging measures. Past studies have established that attachments are essential in the delivery of force and predictability of tooth movement in the case of aligners [9]. Biomechanical studies have also indicated that aligners are capable of providing effective intrusive forces when attention geometry and correct aligner fit are included in the treatment planning [10]. Moreover, the restriction of the size of programmed tooth movement with each aligner has also been indicated to enhance the precision of the attained movements, in line with the staging strategies used in the current study [16]. There was minimal molar extrusion, which showed good posterior vertical control [17]. Aligners have been reported to be in the form of the full-coverage design that acts as a posterior bite-block to decrease the undesirable molar eruption during treatment [8, 18]. The effect of this posterior discussion is especially beneficial in deep bite cases because the extrusion of the molar too much has been identified to result in undesirable facial alterations and loss of stability after treatment [19]. The period of treatment in the current study was similar to the time of treatment in the previous aligner-based studies. The efficiency of treatments with improved aligner materials and computerized planning is proved to be higher, but the necessity of refinement aligners in about a quarter of cases is also consistent with the earlier results that indicated that in many cases complex vertical movements may demand mid-course corrections [14, 20]. Although it is proved that clear aligners are clinically effective with a variety of orthodontic tooth movements, vertical adjustments like intrusion are covered by high treatment planning and can need improvements to demonstrate the best results [21]. New improvements in aligner biomechanics and material characteristics have made the consistency of forces and control of intrusive movements further, leading to an increased level of efficiency regarding deep bite correction [22]. Full-coverage aligner designs have also demonstrated the ability to provide the posterior vertical control that has been reported to makes deep bite correction straightforward without unwanted gains in lower anterior facial height [23]. Compliance by patients has continued to be a key success factor in clear aligner therapy with improper wear time becoming a major draw down of efficacy of aligner-based forces [11]. Though there was no objective measure of compliance in the current retrospective study, the fact that all patients have been successfully treated indicates that they have been adherent. It has been stated that when it comes to future studies, electronic wear-time monitoring systems should be used with the intention of further correlating the compliance with the treatment responses, especially when discussing the cases of the complex vertical tooth movements [24]. The shortcomings of the current research are that it is retrospective, the sample size is moderate and there is no control group with fixed appliances. Though these are some limitations, retrospective clinical studies offer useful real-life evidence and helps in the improvement of clinical decision-making when randomized controlled trials are not available [25]. On the whole, the results of the current paper support the idea that effective deep bite correction based on the use of clear aligner therapy is not only the result of digital planning but also the knowledge and skills of the clinician in applying the orthodontic biomechanics, such as the proper design of attachments, the controlled staging and the proper use of the posterior vertical control [26].

## Conclusion:

Clear aligner therapy can effectively correct deep bite malocclusion when proper biomechanical principles are incorporated into treatment planning. Significant overbite reduction is primarily achieved through controlled maxillary and mandibular incisor intrusion with minimal posterior extrusion. Appropriate attachment design, accurate staging and posterior vertical control are essential for predictable outcomes and successful clinical management.

## Advancement to knowledge:

This study provides recent clinical evidence on deep bite correction using clear aligner therapy. It demonstrates that controlled incisor intrusion is the primary mechanism for overbite reduction with aligners. The findings highlight the importance of attachment design and posterior vertical control in improving predictability. These observations contribute to contemporary biomechanical understanding of aligner-based deep bite management. Clear aligner therapy has been reported to achieve deep bite correction effectively when supported by appropriate attachment design, bite ramps, and staging protocols to enhance predictable incisor intrusion and posterior vertical control.

## Figures and Tables

**Table 1:** Comparison of pre- and post-treatment overbite

**Parameter**	**Pre-treatment (Mean±SD)**	**Post-treatment (Mean±SD)**	**Mean Difference**	**p-value**
Overbite (mm)	5.5±0.7	2.3±0.6	3.2±0.6	<0.001*
*Statistically significant
(paired t-test)

**Table 2:** Changes in vertical tooth position

**Parameter**	**Mean Change (mm)**	**Standard Deviation**
Maxillary incisor intrusion	1.7	0.4
Mandibular incisor intrusion	1.4	0.3
Molar extrusion	0.6	0.2

**Table 3:** Treatment characteristics and mechanical variables

**Variable**	**Observation**
Sample size	30 patients
Mean treatment duration	14.1 ± 2.0 months
Primary bite-opening mechanism	Incisor intrusion
Posterior vertical control	Minimal molar extrusion
Cases requiring refinement	8 (26.7%)
